# Social exchange theory: Systematic review and future directions

**DOI:** 10.3389/fpsyg.2022.1015921

**Published:** 2023-01-12

**Authors:** Rehan Ahmad, Muhammad Rafay Nawaz, Muhammad Ishtiaq Ishaq, Mumtaz Muhammad Khan, Hafiz Ahmad Ashraf

**Affiliations:** ^1^Imperial College of Business Studies, Lahore, Pakistan; ^2^Banking and Finance, University of the Punjab, Lahore, Punjab, Pakistan; ^3^Quaid-i-Azam University, Islamabad, Pakistan; ^4^Management Sciences, University of Central Punjab, Lahore, Punjab, Pakistan

**Keywords:** social exchange theory, reciprocity, workplace relations, evolution of social behaviors, social exchange behavior

## Abstract

Social exchange theory (SET) is one of the most influential theories in social sciences, which has implications across various fields. Despite its usefulness being a typical social transaction, there is a need to look at it from the lens of psychological transactions to further its evolution and to identify future directions. After generally reviewing 3,649 articles from the Social Science Citation Index and Scopus, a total of 46 articles were selected for final review using a comprehensive systematic review approach. We have highlighted the need for further research in psychological transactions, reciprocity principles, exchange relations, and the impact of various factors on the exchange process. Among other exchange rules (social, economic, and psychological) and transactions (social, economic, and psychological), this research provides an elevation platform for the less explored exchange rules in psychological transactions. Among other theories in the social sciences, social exchange theory is a theory that shadows many other theories under its umbrella.

## 1. Introduction

Social exchange theory (SET) is one of the gold standards to understand workplace behavior (Cropanzano and Mitchell, 2005). It is such a common phenomenon that is deeply inculcated in our daily lives. Exchanges are not limited to the organizations but extended to our family, friends, and relatives, and that too on a subtle basis. Cropanzano et al. (2017) defined the SET as (i) an initiation by an actor toward the target, (ii) an attitudinal or behavioral response from the target in reciprocity, and (iii) the resulting relationship. Relationships in the corporate world today are becoming increasingly complex (Chernyak-Hai and Rabenu, 2018). Hence, there is a need to update SET with the increasing complexity of how organizations operate and how employees behave (Cooper-Thomas and Morrison, 2019).

Rooted back in the 1920s (Malinowski, 1922; Mauss, 1925), social exchange theory has implications across various fields like social psychology (Homans, 1958; Thibault and Kelley, 1959; Gouldner, 1960), sociology (Blau, 1964), and anthropology (Firth, 1967; Sahlins, 1972). It was Homans (1958), who, for the first time, proposed the idea of “Social behavior as exchange” in the literature, and he further evolved this idea into its elementary forms in 1961. Thibault and Kelley (1959) proposed the converging notion of the “social psychology of groups.” Blau (1964) further evolved this idea by presenting the concept of “exchange and power,” which refers to the ability of one party to influence another party to do something. Blau highlighted the economic orientation of the theory, while Homans lodged more upon psychological orientation, that is, instrumental behavior. According to a significant contribution by Blau (1964) in literature, social exchange conceived here is limited to actions that are contingent on rewarding reactions from others, and exchange behavior means voluntary actions of individuals that are motivated by the returns they are expected to bring.

Homans (1969) further evolved his study in SET, incorporated sociology, and behavioral psychology concepts and stressed the need for further research on the subject, while Anderson et al. (1969) reinforced the economic implications of the theory. Goode proposed the idea that the role theory and exchange theory were convergent to one another in 1973. Emerson (1976a) suggested that SET is not a theory but a frame covering many theories under its shadow. Other areas analyzed under the light of SET include commitment (Bishop et al., 2000), organizational citizenship behaviors (Organ, 1990), supervisory and organizational support (Ladd and Henry, 2000), and justice (Tepper and Taylor, 2003). Mitchell et al. (2012) proposed the idea of a social life cycle that refers to events/transactions between parties.

Cropanzano et al. (2017) proposed that the action of the first actor is termed initiating action and is divided into positive and negative ones. Positive initiating actions include justice (Cropanzano and Rupp, 2008) and organizational support (Riggle et al., 2009), and negative actions may consist of incivility (Andersson and Pearson, 1999; Pearson et al., 2005), abusive supervision (Tepper et al., 2009), and bullying (Rayner and Keashly, 2005). The resulting response from the target can be classified as behavioral and relational. Subsequently, successful exchanges eventually transform a preliminary economic exchange into a social exchange relationship (Cropanzano et al., 2017). Lyons and Scott (2012) proposed the idea of “homeomorphic reciprocity” which refers to the ability of an employee to receive help or harm shall depend upon the extent to which that employee engages in benefit and harm. Additionally, the behaviors exchanged between an employee and a given coworker should be equivalent, such that engaging in help, but no harm, is associated with receiving support, and engaging in harm, but not help, is associated with receiving harm.

Having such broad applications, according to the study of Cropanzano and Mitchell (2005), the core ideas that comprise SET have yet to be adequately articulated and integrated. Researchers further concluded that SET is a broad framework that can describe almost any finding (Sharpley, 2014; Cropanzano et al., 2017). Such broadness shows the presence of flexibility and variety in SET consequently. At the same time, various researchers embark upon social and economic transactions and exchanges in SET. Based on the call of Cropanzano et al. (2017), this article aims to investigate more upon inactive exchanges, which we termed as psychological exchanges. Active exchanges are visible, while inactive exchanges are less visible and are positive (withholding undesirable behavior) as well as negative (withholding desirable behavior). The shadow nature of the inactive exchanges can turn out to be more damaging for the organization as it is difficult to trace. Moreover, on the basis of the rules of reciprocity, usually more behaviors are inactive and destructive rather than inactive and constructive. Hence, these inactive exchanges are important to explore for a better understanding of SET.

Moreover, building on the definition of SET by Cropanzano et al. (2017), this article further proposes that initiating action, which is found to be explicit, can be implicit, such as a feeling (positive or negative), and can be an outcome of someone’s achievement (feeling jealousy at the promotion of a coworker, a psychological exchange). This article comprehensively outlines the evolution of SET and introduces a new dimension in social exchange relationships and ultimately provides future direction for further research.

## 2. Methods

To understand the social exchange theory and its evolution, one should begin by identifying the roots of the concept and elaborate on the differences and commonalities in the work of various authors in academic literature. The literature highlights different definitions, rules, approaches, and dimensions in the evolution of SET. To understand the concept of SET, three different areas are acknowledged using content analysis of 3,221 articles indexed in the ISI Web of Knowledge and Scopus. The areas are (1) basic concepts of SET as they evolved, (2) exchange rules that govern social exchanges, and (3) evolving dimensions of the exchange relationships. The theoretical framework used in this article is in line with the study of Yadav (2014) and MacInnis (2011), where they propose to differentiate and assimilate particular conceptual goals. We searched the ISI Web of Knowledge and Scopus along with Social Sciences Citation Index from 1920 to 2020 because the concept of SET goes back to 1920.

Search results from the Sciences, Arts, and Humanities Citation Index were eliminated, and the results were filtered for Business and Management, Social Sciences, and Psychology. We used multiple keywords in the ISI search engine in *the topic* field using a complete list of possibilities including “social exchange theory,” “exchange relationships,” “evolution of social exchange theory,” and “exchange relations.” These searches returned highly significant empirical and conceptual references (*n* = 3,221; Scopus = 1954 and ISI Web of Knowledge = 1,267). After the search, duplicate articles (*n* = 1,526) in both databases were deleted.

In the next step, conceptual and empirical articles on SET were separated and analyzed to identify and track evolution patterns, and empirical articles with no theoretical contribution (*n* = 1,202) were excluded. In the next phase, those articles were eliminated through contextual analysis that had meager theoretical contributions or available models’ allowance (*n* = 446). The purpose of this article was to classify the evolution of SET to propose needed contributions. Hence, after excluding empirical articles and literature reviews with no progression in SET, we ended up with 47 articles (Table 1). Out of the articles that were selected for the final review, two of them were published in the decade between 1920 and 1930, three between 1951 and 1960, five between 1961 and 1970, nine between 1971 and 1980, four between 1981 and 1990, eight between 1991 and 2000, 10 between 2001 and 2010, and nine between 2011 and 2020.

**Table 1:** Evolution of social exchange theory.

Year	Author(s)	Evolution
1920–1930	Malinowski (1922)	The circulating exchange of’ valuables in the Archipelagoes of Eastern New Guinea.
	Mauss (1925)	Forms and functions of exchange in Archaic Societies.
1951–1960	Homans (1958)	Social behavior as exchange psychological orientation.
	Thibault and Kelley (1959)	The social psychology of groups.
	Gouldner (1960)	Incorporated types of reciprocity (transaction, belief, moral norm) in the concept of SET.
1961–1970	Blau (1964)	Exchange and power economic orientation.
	Firth (1967)	The implication of SET in anthropology.
	Homans (1969)	Incorporated the concepts of sociology and behavioral psychology.
	Gergen (1969)	Transactions mean interdependent exchanges.
	Anderson et al. (1969)	Reinforced the economic implications of SET.
1971–1980	Meeker (1971)	Proposed six exchange rules as competition, group gain, status consistency, altruism, rationality, and reciprocity.
	Sahlins (1972)	Presented comparison of stone age economics with SET and highlighted implications of SET in anthropology.
	Goode (1973)	Role theory and exchange theory are convergent to one another.
	Emerson (1976a)	SET is not a theory but a frame that covers many theories under its shadow.
	Foa and Foa (1974)	Classifications of exchange resources as status, information, goods, love, money, and services.
	Emerson (1976b)	Exchange relationships are based on the rules of the exchange.
	Clark and Mills (1979)	Classification of individuals based on the degree of reciprocity.
	Foa and Foa (1980)	Classification of exchange resources in two dimensions as economic (tangible) and socioemotional (symbolic).
	Lerner (1980)	The idea of a “just world” in exchange relationships.
1981–1990	Mills and Clark (1982)	Proposed competition and communal exchange relationships.
	Cook et al. (1983)	Concept of terms and rules in social exchange to reach interdependent goals.
	Folger and Konovsky (1989)	SET is beyond the rules of transactions and benefits.
	Organ (1990)	Organizational citizenship behavior in light of SET.
1991–2000	Cropanzano and Baron (1991)	Concept of seeking revenge in an exchange relationship.
	Martin and Harder (1994)	Tangible and symbolic dimensions of exchange resources are based on different exchange rules.
	Molm (1994)	Interdependence in exchanges overcome risks and supports cooperation.
	Chen (1995)	Dimensional classification of exchange resources.
	Batson (1995)	Altruism as an exchange rule.
	Bies and Tripp (1996)	SET concerning justice can reduce destructive behavior in people.
	Bishop et al. (2000)	Organizational commitment in the light of SET.
	Ladd and Henry (2000)	Supervisory and organizational support.
2001–2010	Tsui and Wang (2002)	SET is a moral norm.
	Rhoades and Eisenberger (2002)	Explored exchange relationships as POS and LMX
	Rupp and Cropanzano (2002)	Mediating role of social exchange relationships in predicting workplace outcomes.
	Wang et al. (2003)	SET is embedded in humans universally.
	Molm (2003)	Concept of negotiated exchanges.
	Tepper and Taylor (2003)	Organizational justice in the light of SET.
	Eisenberger et al. (2004)	Concept of positive and negative reciprocity.
	Coyle-Shapiro and Conway (2004)	Employment relationship through the lens of SET.
	Cropanzano and Rupp (2008)	Justice as positive initiating action.
2010–2020	Mitchell et al. (2012)	The social life cycle refers to events/ transactions between parties.
	Lyons and Scott (2012)	Homeomorphic reciprocity.
	Sharpley (2014)	SET as a broad framework that can describe almost any findings
	Ko and Hur (2014)	Rules of exchange introduced in the literature.
	Methot et al. (2016)	The concept of multiplex relations in social exchanges was introduced, which includes formal and informal relations.
	Cropanzano et al. (2017)	Redefined SET as (i) an initiation by an actor toward the target, (ii) an attitudinal or behavioral response from the target in reciprocity, and (iii) the resulting relationship.
	Cropanzano et al. (2017)	Reciprocity happens both explicitly and implicitly. Concept of transactional chains. Addition of activity dimension.
	Cooper-Thomas and Morrison (2019)	Implications of SET in complicated organizational settings.
	Hossen et al. (2020)	Exchange relationships are the results of mutual benefits.

Source: Authors generated this table from searches on the ISI Web of Knowledge and Scopus. All citations in the table are listed in the reference list.

## 3. Key ideas of set

We shall begin by curating the underlying ideas which comprise SET which involve rules and norms of exchange, resources exchanged, and resulting relationships (Methot et al., 2016; Cropanzano et al., 2017). A comprehensive snapshot of key ideas related to SET across the years is presented in Table 2.

**Table 2:** Key ideas related to SET.

Key ideas	Authors
Rules and norms of exchange	Cropanzano and Mitchell (2005)
	Emerson (1976b)
	Ko and Hur (2014)
Reciprocity rules	Gouldner (1960)
	Molm (1994)
	Molm (2003)
	Lerner (1980)
	Bies and Tripp (1996)
	Cropanzano et al. (2017)
Rules of exchange	Cook et al. (1983)
	Molm (2003)
	Meeker (1971)
	Batson (1995)
Resources of exchange	Foa and Foa (1974)
	Foa and Foa (1980)
	Cropanzano and Mitchell (2005)
Social exchange relationships	Shore and Coyle-Shapiro (2003)
	Blau (1964)
	Mills and Clark (1982)
	Cropanzano and Mitchell (2005)
	Eisenberger et al. (2004)
	Molm (2003)
	Eisenberger et al. (2004)
	Methot et al. (2016)
	Cooper-Thomas and Morrison (2019)
	Cropanzano et al. (2017)
Transactions and exchange relationships	Cropanzano and Mitchell (2005)
	Cropanzano et al. (2017)
	Cropanzano et al. (2017)

All citations in the table are listed in the reference list.

### 3.1. Rules and norms of exchange

One of the fundamental pillars of SET is that commitment, loyalty, and trust are upshot of evolving relationships with time (Cropanzano and Mitchell, 2005). This pillar demands that parties must show compliance toward specific rules (i.e., rules of exchange). According to Emerson (1976b), such rules form a normative definition of the participants in an exchange relation adopted. Hence, such an exchange principle facilitated avenues for researchers in organizational behavior to further their work (Cropanzano and Mitchell, 2005). Most management research is focused on the potential of reciprocity. Ko and Hur (2014) stressed that other rules of exchange exist that the researchers do not sufficiently explore. This article, therefore, analyzes reciprocity and other less-explored exchange rules.

#### 3.1.1. Reciprocity rules

Gouldner (1960) made a significant contribution to the literature by outlining rules of reciprocity as (a) transaction, (b) belief, and (c) moral norm. The transaction, according to Gouldner (1960), meant interdependent (both dependent on one another) exchanges, and this idea was then reinforced by Molm (1994). A reciprocal exchange due to interdependence curbs risks and supports cooperation, according to Molm (1994), and does not include pronounced bargaining (Molm, 2003). As per the idea, the exchange is a continuous cycle where one party makes a move, and the other reciprocates, and it begins a new cycle of exchanges (Cropanzano and Mitchell, 2005). Suffice it to say that there is a vast literature on the interdependence of exchange and transaction, and reviewing that literature would bypass the scope of this article.

The second rule of reciprocity, that is, reciprocity as belief, revolves around cultural orientation (Gouldner, 1960). This orientation is in line with the idea of karma: You get what you deserve. The idea of a “just world” proposed by Lerner (1980) is consistent with this type of reciprocity. Furthermore, it reduces destructive behavior in people (Bies and Tripp, 1996). Gouldner (1960) speculated that reciprocity is a moral norm and is embedded in humans universally (Tsui and Wang, 2002; Wang et al., 2003). Nevertheless, it is important to note that humans are different, and the way they reciprocate depends heavily on their cultural and individual differences (Parker, 1998; Coyle-Shapiro and Neuman, 2004).

Social psychologists such as Clark and Mills (1979) and Murstein et al. (1977) proposed classifications of individuals based on the degree of reciprocity. They termed the classification “high exchange orientation” (those who readily reciprocate) and “low exchange orientation” (those who do not return or reciprocate less). This unleashed avenues for further research in management as scholars worked on various avenues such as absenteeism (Eisenberger et al., 1986), felt obligation (Eisenberger et al., 2001), citizenship behavior (Witt, 1991), satisfaction and training (Witt and Broach, 1993), performance (Orpen, 1994), union support (Sinclair and Tetrick, 1995), job commitment and satisfaction (Witt et al., 2001), and organizational politics (Andrews et al., 2003).

Many researchers, including Uhl-Bien and Maslyn (2003) and Eisenberger et al. (2004), further classified reciprocity as positive (reciprocating favorable treatment) and negative (reciprocating unfavorable treatment). Cropanzano and Mitchell (2005) called for further investigation into the impact of social exchanges on organizational relationships and also proposed the need for research in unexplored areas such as coworkers, supervisors, and outsiders. Building on previous literature, Cropanzano et al. (2017) proposed that people may not reciprocate the way they wish due to various uncontrollable factors (the presence of inadequate supervision and fewer turnover intentions due to a bad economy). Cropanzano et al. (2017) further added to the literature of SET that reciprocity happens, both explicitly (active exchanges) and implicitly (inactive exchanges). Both forms communicate in exciting ways. For instance, an employee will have high work deviance (implicit) but will not leave the job due to a lousy economy in terms of inactive exchanges (explicit). Moreover, Greco et al. (2019) investigated the reciprocity of negative work behaviors between two parties and reported that negative work behaviors are returned on the similar intensity and capacity between the two parties.

Individual differences in reciprocity are presented in chronological order in Appendix 1.

#### 3.1.2. Negotiated rules and other exchange rules

Parties in a social exchange may negotiate terms or rules to reach interdependent goals (Cook et al., 1983). There is significant literature on the comparison of reciprocal and negotiated exchanges (Molm, 2003). Key findings suggest that better work relations are the outcome of reciprocity than negotiations. Exchange rules other than reciprocity and negotiation gained more attention in literature from sociology and anthropology researchers than from management researchers (Fiske, 1991). One notable study by Meeker (1971) proposed six exchange rules: competition, group gain, status consistency, altruism, rationality, and reciprocity.

According to Meeker (1971), rationality is a thought process asking for justification for various actions taken by a person according to his preferences. Altruism is about being compassionate and kind, where the good of others is essential, even at the cost of ourselves. This sounds uncanny, but the literature supports the take of Meeker (1971) on altruism as an exchange rule (Batson, 1995). Group gain refers to contributions, and everybody takes (benefits) according to their desire. Group gain omits the idea of interpersonal exchanges and extends the horizon toward group exchanges. Status consistency is also called rank equilibrium, where the disunion of benefits depends upon one’s standing in a social group. Lind (1995) experimented with and supported this exchange rule.

Competition is directly the opposite of altruism, where altruism is about benevolence, and competition is about self-seeking behavior (Meeker, 1971). This opened doors for research on modern-day variables in organizational behavior such as workplace envy (Ahmad et al., 2020), organizational politics, and political skills. The study of Meeker (1971) also strengthened the idea of seeking revenge in an exchange relationship (Cropanzano and Baron, 1991; Turillo et al., 2002). A great deal of literature exists on reciprocity as a rule of exchange. Still, there are other rules, such as group gain, status consistency, competition, altruism, and rationality, which require attention and investigation. Exploring these will open doors to fathom the process of social exchanges, which is still unexplored to a great deal (Cropanzano and Mitchell, 2005). Moreover, there is a possibility that multiple exchange rules are employed at once.

### 3.2. The resources of exchange

Foa and Foa (1974) proposed classifications of exchange resources as status, information, goods, love, money, and services. These resources can be termed as benefits that a person seeks in social exchange and can be further classified into two dimensions economic (tangible) and socioemotional resources (symbolic) (Foa and Foa, 1980). Both dimensions work on different exchange rules (Martin and Harder, 1994). Resources and their dimensional classification are still not sufficiently explored and are open for further investigation. Furthermore, the relationship between types of resources and the type of relationship is also an open area for research (Cropanzano and Mitchell, 2005).

### 3.3. Resulting relationships: Social exchange relationships

Workplace relationships are the most explored area in management research (Coyle-Shapiro and Conway, 2004). However, much of the research on exchange relations is done in employer–employee relations (Blau, 1964). His study is based on the premise that much of social relations are based on unspecified obligations. This makes the relations more casual while successful exchanges are based on the commitment between parties. Blau (1964) also considered relations as transactions. Mills and Clark (1982) further contributed to the literature by proposing two types of exchange relationships. One is exchange relations based on competition, and the others are communal relations based on benevolence. Organ (1990) found that SET is beyond the rules of transactions and benefits, and this extended the scope for further research in SET.

Suffice it to note that relations are termed as associations between partners, which can be institutions and individuals (Cropanzano and Mitchell, 2005). Although much of the research is done on exploring the relations between institutions and individuals such as employing organizations (Moorman et al., 1998), customers (Houston et al., 1992), and suppliers (Perrone et al., 2003), the literature is comparatively silent on the area of individual relationships in an organizational setting such as peer relations. Notable work in management is done in terms of exchange relationships which are perceived organizational support (POS), Leader–Member Exchange (LMX; Eisenberger et al., 2004), support to commitment (Eisenberger et al., 1990), team support and organizational support (Bishop et al., 2000), supervisor support (Masterson et al., 2000), and trust (Dirks and Ferrin, 2002).

It is also important to state that relationships develop over time ranging from premature relations (Molm, 2003) to mature ones (Eisenberger et al., 2004). Building on the premise of increasingly complex relationships at the workplace, Methot et al. (2016) introduced the term “multiplex” relations at the workplace, which include both formal (work-related) and informal (friendship) elements. Such relations cover both positive (e.g., emotional support) and negative (e.g., emotional exhaustion) aspects. Cooper-Thomas and Morrison (2019) identified that it is not clear how SET might apply in conditions where positive and negative exchanges are simultaneously taking place.

As multiple behaviors are exchanged in the workplace, Cropanzano et al. (2017) tossed the term “transactional chains” through which relationships are developed over time through various exchanges. If we want to understand the form of a relationship, we must understand the principal transaction of resources responsible for a particular relationship. Building on the need to understand SET in further detail highlighted by Cropanzano et al. (2017) and Cooper-Thomas and Morrison (2019), we shall elaborate on the transactions and resulting exchange relationships.

#### 3.3.1. Transactions and exchange relationships

Cropanzano and Mitchell (2005) highlighted two distinguishing aspects of relationships in the literature. One aspect is a relationship as the series of interdependent transactions transpires to interpersonal attachment, which is a relationship. Alternatively, another element is the interpersonal relationship that originates from interdependent exchanges. It is essential to distinguish the relationship from the transaction process because of its interchangeability. The nature of the relationship between two parties is dictated by the process of exchange or the benefits they exchange between them. When a series of exchanges happen, it becomes rather challenging to find which exchange caused the relationship.

**Figure 1:**
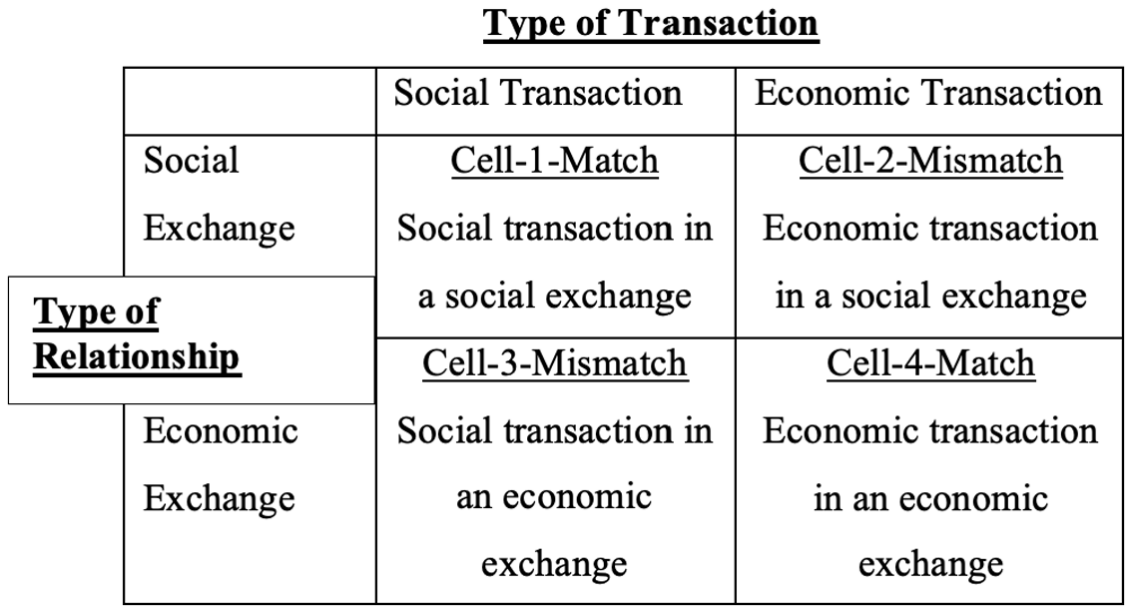
Relationships of transactions in exchanges.

Researchers separated the form of exchange from the exchange relationship presented in Figure 1. Cells 1 and 4 can be termed *matches* as the form of transaction coinciding with the relationship. The situation in Cell 2, where the social exchange relationship coincides with the economic transaction, could reap both risks and rewards. For instance, social relations are at greater risk in economic exchanges, and hence, economic exchanges can pose a more significant threat to relationships (clashes in the inheritance among family members). Alternatively, while considering rewards, greater trust and stronger relationships can be an outcome for such exchanges (father giving money to son and not asking for details). Cell 3 presents the unusual case of emotional labor where employees from the hospitality industry or health workers attend to the emotional needs of their clients or patients for money (economic transaction).

People working in mental asylums display such behaviors to fulfill their professional duties. Similarly, people working in the hotel and hospitality sector are expected to be friendly with their clients. It is tricky and stressful to share such emotions with others, expected to be family members or other loved ones. While keeping in view, the vagueness of the concept of relationships in SET, Cropanzano and Mitchell (2005) highlighted two distinct conceptual dimensions of the relationship. One is a sequence of inter-related exchanges, and the other is relationships as an outcome of codependent exchanges. These are termed transactional and interpersonal relationships in the literature. When relationships seem to transcend over one another, it becomes more challenging to define them. It is essential to understand that two different things can be exchanged through various means among two different parties.

## 4. Discussion: Beyond socio-economic transactions

**Figure 2:**
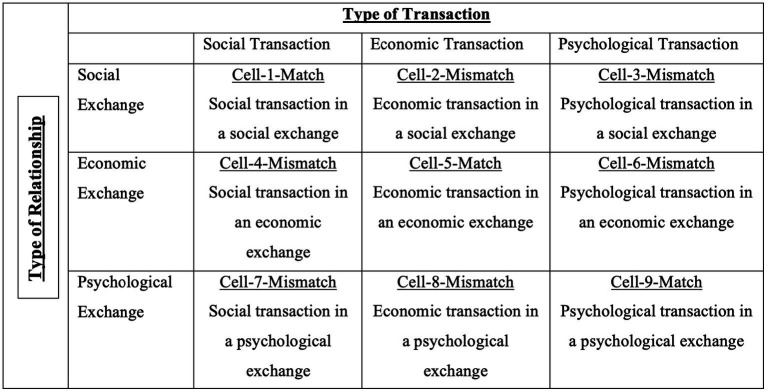
Proposed model of transactions and exchanges.

Building on the aforementioned model, we propose that while looking beyond the lens of social and economic transactions and exchanges, relationships are also psychological. This premise is based on the idea of implicit or inactive exchanges proposed by Cropanzano et al. (2017). The concept of psychological capital (Luthans et al., 2007) also supports this idea, and exchanges in such relations can be termed psychological exchanges. Referring to Figure 2, Cells 1, 2, 4, and 5 are similar to Cells 1, 2, 3, and 4 in Figure 1. Unique cells in Figure 2 are Cells 3, 6, 7, 8, and 9. Cell 9 is a matching cell coinciding psychological transaction with a psychological exchange relationship. Let us first hone ourselves with the idea of psychological transactions.

To start with, psychological transactions are usually inactive exchanges. From this dimension, it sounds easier to draw that psychological exchange relations are inactive relations, which is incorrect. Psychological relations are based on the *understanding* between the two parties. From “understanding,” it means how well parties in a social exchange know each other. This, according to the empirical evidence, indicates that parties develop relationships after being involved in a series of exchanges, and eventually, they develop a relationship so good that they can understand each other on psychological fronts as well. Nevertheless, this is not true as Cell 3 clarifies that psychological transactions may not necessarily occur in every social relationship.

Putting it further, it is challenging to find like-minded people with whom our mental chemistry aligns. Referring to Cell 6, which draws a dimension about the psychological transaction in an economic relationship, it is evident that psychological transactions do occur during economic relations, but such transactions are usually dubious. The reason for this is that such transactions are generally solitary and not dyad. Due to this attribute, past researchers called them inactive exchanges. Cell 7 presents the case of clinical psychology, where psychiatrists develop a psychological relationship with patients or subjects in a social setting.

Similarly, researchers also fall into this category to build empathy through social transactions to collect data. Cell 8 is similar to Cell 7, and diffusion can be drawn in the *intent*. Cell 7 refers to social welfare, while Cell 8 refers to economic return. If a researcher is working on a social problem or aiming to find a cure for a disease such as COVID-19 without aiming for lucrative gains, he will fall into Cell 7. On the contrary, if Toyota launches an electric vehicle or Philips launches a light bulb that consumes less electricity with a pure aim to sell these products to those consumers who want to save on their gas or electricity bills, they would fall in the Cell 8. If a transaction is taken as a relationship, then successful exchanges will be accepted as its outcome. It works both ways, from transactions in relations to relations in transactions (Figure 2).

To explain how psychological transaction and psychological exchange relations work, the model by Foa and Foa (1980) comes to rescue from the literature. This model aligns a variety of resources according to different relationships, such as causal and universal. Causal relations complement universal resources, while intimate relations complement particularistic resources. Interestingly, a universal benefit paves the way for particularistic use, and this is how relationships become an outcome of reciprocal exchanges. Hence to understand this concept of exchange, we need to further our understanding related to exchange models. As to further contribution to SET literature, two models are proposed below to provide conceptual support to the dimensions of psychological transactions and psychological exchange relationships.

### 4.1. Nature of relations affects the psychological exchanges

Eisenberger et al. (2001) suggested that employees in an organization can exchange commitment in the reciprocation of organizational support. This finding allowed us to build our argument that the nature of relations between parties who participate in an exchange process can affect psychological exchanges. In other words, the closer the relationship between the two parties (pluralistic exchanges), the more there will be psychological exchanges. The key term to note here is “close,” which means seeing someone like peers or classmates every day. Furthermore, the achievement of a friend or classmate who went abroad will affect us less than someone we see every day.

This happens because of the social comparison we do with people near us. Hence, social distance or space between the parties does affect the relationship between them. Moreover, such a relationship will directly impact the intensity or type of psychological exchanges between them. It is important to note that not only the positive relationship enables the possibility of psychological exchanges, but it can also have a similar impact in terms of hostile relations as well. Similarly, a positive relationship does not necessarily mean that there will be only complementary psychological exchanges; negative psychological exchanges can also occur. For instance, you are feeling jealous about the good grades of your best friend. But such a psychological exchange would be different from the one you would have against someone in the class you dislike.

### 4.2. Psychological exchanges affect the nature of relations

Psychological exchanges in an organization are not a one-time thing but a continuous process like climbing a ladder. In other words, it constitutes a series of transactions between parties in a work setting. Hence, the output of a transaction today will form the psychological resource (both positive and negative) that can be exchanged tomorrow or anytime in the future. Therefore, psychological exchanges can form the basis of relationships between the parties. Positive psychological exchanges become a reason for positive relations, and negative psychological exchanges can cause negative associations (rivalry—usually between coworkers).

It is imperative to note that the exchange timing plays a significant role in forming the relations between parties. This timing of exchange dimension is coherent with the model of LMX development proposed by Uhl-Bien and Maslyn (2003). This model suggests that leaders and members start their relationship journey by testing one another in terms of obligations, and the quality of relations depends upon the reciprocity of commitments. Suffice it to say that positive psychological exchanges result in the exchange of positive psychological resources. Similarly, negative psychological exchanges result in the exchange of harmful psychological resources, which impact resulting relationships.

## 5. Recommendations and future directions

Having its roots in the 1920s (Malinowski, 1922; Mauss, 1925), the scope and foundations of SET are yet to be sufficiently explored. Management researchers have characteristics of a variety and multiple applications and are doing injustice with this theory in two ways. First, they lack the indulgent understanding of ideas that set the foundations of SET. Second, limited avenues are being explored in the research as reciprocity principles and economic orientation of SET. Cropanzano et al. (2017) investigated that people may not reciprocate according to their wishes due to certain uncontrollable factors. Cooper-Thomas and Morrison (2019) identified that it is not clear how SET might apply in conditions where positive and negative exchanges are simultaneously taking place.

We believe that this article shall help address both shortfalls as it adopts a meek way to outline the evolution of SET and identify essential areas where researchers can direct their future efforts. This article shall help dramatically evolve the theory by revising existing concepts, orientations, and forming new ones. According to Eisenberger et al. (1986) and Graen and Scandura (1987), SET comprises two types of social exchanges. First is perceived organizational support (POS) that emphasizes employee–organization exchange relationships.

The second is the exchange between the leader and member, which elaborates on the interaction between the supervisor and the employee through the exchange of resources (Lee and Duffy, 2019). In both types of exchanges, resulting relationships work as a cynosure of the exchange process. Consequently, the understanding of SET would remain meager if we could not hone the idea of exchanges and resulting relationships. This article pronounced the social and economic transactions and exchanges from the literature and proposed a new *psychological* dimension with empirical and conceptual justifications. This idea is similar to Cropanzano et al. (2017), who introduced the concept of active and inactive exchanges, which revolutionized the whole notion of SET.

According to these dimensions, exchanges in organizational settings happen both explicitly (active exchanges) and implicitly (inactive exchanges). More notably, in the presence of uncontrollable factors, employees will still reciprocate but implicitly. The idea of how employees may get involved in inactive exchanges, even in the absence of uncontrollable factors, is another open avenue for future research. Take an instance of workplace envy: Workplace envy is an inactive exchange (beneficial or costly) of an employee in an organizational setting. It is a feeling that could be visible through active exchanges.

Building on these developments, this study proposes that social exchange may not necessarily be dyadic; it can be individualistic or monotonous where an employee feels on his own. The role of psychological transactions and resulting psychological exchange relationships can be understood from a case as simple as an employee feeling jealous about the achievement of a coworker. This dimension is inevitable, and it nulls the first part of the definition of SET, that is, *initiation by an actor*. This is because no one is initiating, and an employee envies himself or inactive exchange is taking place. Future studies should help to unveil this process of SET in further detail. Moreover, the current study focused on organizational exchanges and resulting relationships, and future research efforts can be directed toward social exchanges among family, friends, and relatives to improve the understanding and scope of SET.

It is also pertinent to note that negative emotions and feelings may be controlled through specific skills such as political skills and social skills. While there is much research on social exchanges in organizational relationships, areas of coworkers, supervisors, and outsiders are yet to be sufficiently explored. Moreover, Foa and Foa (1980) proposed classifications of exchange resources as status, information, goods, love, money, and services. These resources can be further classified into two dimensions economic (tangible) and socioemotional resources (symbolic). On account of social exchange relationships, much of the research is done on exploring relations among institutions and individuals (Moorman et al., 1998), customers (Houston et al., 1992), and suppliers (Perrone et al., 2003), whereas literature is comparatively silent on the area of interpersonal relationships in an organizational setting.

There are exchange rules beyond reciprocity, exchange resources above money, and trust, and there are types of relationships other than social, economic, and psychological that need to be explored. These resources and their impact on social relationships are also unexplored areas asking for attention from the researchers. In addition to the above discussion, the following points can pave the way for a better understanding of SET and future research.

- It is unnecessary for a social exchange process that a positive initiating action would generate a positive response.
- Positive initiating action may not form a positive relationship.
- Positive initiating action may not always form a positive relationship, and it can be negative too.
- With changing workplace landscape, relationships are becoming increasingly complex in modern organizations; hence, relations are increasingly affecting the modern exchange process.
- An implicit initiating action can cause implicit and explicit behavioral responses.
- In some social instances, such as envy, the exchange process can be hidden, and hence, an actual exchange process could be altered with a fabricated exchange process.

## 6. Conclusion

While SET is evolving, it is inviting researchers to explore various related avenues. Thus, a broad theory that can shadow many other theories under its umbrella can describe multiple social phenomena. This article provided comprehensive commentary about how SET evolved and recent progressions, and it also provides fruit of thought on the psychological dimension that exists under the disguise of inactive exchanges. Beyond social and economic transactions, the idea and implications of psychological transactions are proposed in this article. Based on the idea of inactive exchanges, it is also proposed that other than reciprocity, other less explored exchange rules are dominant in psychological transactions.

## Data availability statement

The original contributions presented in the study are included in the article/supplementary material, further inquiries can be directed to the corresponding author.

## Author contributions

RA and MN: concept development and systematic review strategy and final write-up. MI and MK: downloading and reviewing manuscript to be selected for the final review. HA: language of the manuscript, bibliography, and final formatting and review. All authors contributed to the article and approved the submitted version.

## Conflict of interest

The authors declare that the research was conducted in the absence of any commercial or financial relationships that could be construed as a potential conflict of interest.

## Publisher’s note

All claims expressed in this article are solely those of the authors and do not necessarily represent those of their affiliated organizations, or those of the publisher, the editors and the reviewers. Any product that may be evaluated in this article, or claim that may be made by its manufacturer, is not guaranteed or endorsed by the publisher.

## Figures and Tables

**Table:** 

Year	Author(s)	Exchange orientation
1986	Eisenberger et al. (1986)	Exchange ideology
1987	Eisenberger et al. (1987)	Reciprocation ideology
1990	Witt and Wilson (1990)	Exchange ideology
1991	Witt (1991)	Exchange ideology
1992	Witt (1992)	Exchange ideology
1992	Cotterell et al. (1992)	Reciprocation wariness creditor ideology
1993	Witt and Broach (1993)	Exchange ideology
1994	Orpen (1994)	Exchange ideology
1995	Sinclair and Tetrick (1995)	Exchange ideology
1999	Lynch et al. (1999)	Reciprocation wariness
2000	de Ruyter and Wetzels (2000)	Relational exchange orientation
2000	Ladd and Henry (2000)	Exchange ideology
2001	Witt et al. (2001)	Exchange ideology
2001	Perugini and Gallucci (2001)	Personal norm reciprocity
2001	Eisenberger et al. (2001)	Exchange ideology
2003	Shore and Coyle-Shapiro (2003)	Reciprocity norm acceptance
2003	Gallucci and Perugini (2003)	Personal norm of reciprocity
2003	Andrews et al. (2003)	Exchange ideology
2004	Coyle-Shapiro and Neuman (2004)	Exchange ideology creditor ideology
2004	Eisenberger et al. (2004)	Positive norm of reciprocity Negative norm of reciprocity

All citations in the table are listed in the reference list.

## Appendix

Appendix 1 Studies examining individual differences in reciprocity.
